# The prFMNH_2_-binding chaperone LpdD assists UbiD decarboxylase activation

**DOI:** 10.1016/j.jbc.2024.105653

**Published:** 2024-01-13

**Authors:** Deepankar Gahloth, Karl Fisher, Stephen Marshall, David Leys

**Affiliations:** Manchester Institute of Biotechnology, University of Manchester, Manchester, UK

**Keywords:** UbiD decarboxylase, prFMN, chaperone, oxidative maturation, cofactor incorporation

## Abstract

The UbiD enzyme family of prenylated flavin (prFMN)-dependent reversible decarboxylases is near ubiquitously present in microbes. For some UbiD family members, enzyme activation through prFMNH_2_ binding and subsequent oxidative maturation of the cofactor readily occurs, both *in vivo* in a heterologous host and through *in vitro* reconstitution. However, isolation of the active *holo*-enzyme has proven intractable for others, notably the canonical *Escherichia coli* UbiD. We show that *E. coli* heterologous expression of the small protein LpdD—associated with the UbiD-like gallate decarboxylase LpdC from *Lactobacillus plantarum*—unexpectedly leads to 3,4-dihydroxybenzoic acid decarboxylation whole-cell activity. This activity was shown to be linked to endogenous *E. coli ubiD* expression levels. The crystal structure of the purified LpdD reveals a dimeric protein with structural similarity to the eukaryotic heterodimeric proteasome assembly chaperone Pba3/4. Solution studies demonstrate that LpdD protein specifically binds to reduced prFMN species only. The addition of the LpdD–prFMNH_2_ complex supports reconstitution and activation of the purified *E. coli apo*-UbiD *in vitro*, leading to modest 3,4-dihydroxybenzoic acid decarboxylation. These observations suggest that LpdD acts as a prFMNH_2_-binding chaperone, enabling *apo*-UbiD activation through enhanced prFMNH_2_ incorporation and subsequent oxidative maturation. Hence, while a single highly conserved flavin prenyltransferase UbiX is found associated with UbiD enzymes, our observations suggest considerable diversity in UbiD maturation, ranging from robust autocatalytic to chaperone-mediated processes. Unlocking the full (de)carboxylation scope of the UbiD-enzyme family will thus require more than UbiX coexpression.

The UbiD family of reversible decarboxylases is ubiquitous in microbes, catalyzing C^*sp2*^-H + CO_2_/C^*sp2*^-CO_2_H interconversion on unsaturated or (hetero)aromatic substrates (1). These enzymes depend on the prenylated flavin (prFMN) cofactor for activity, requiring the presence of the flavin prenyl transferase UbiX to convert reduced FMNH_2_ to the reduced prFMNH_2_ (2) (Fig. 1). The UbiX product prFMNH_2_ requires a two-electron oxidative maturation to the active iminium form (prFMN^iminium^) to support UbiD catalysis (2). When the cofactor is exposed to oxygen, either free in solution or in context of the UbiX–product complex, one-electron oxidation occurs readily (2). The latter leads to formation of the stable purple-colored prFMN radical that is unable to support UbiD activity or readily undergo further oxidation to the required prFMN^iminium^. For the model UbiD enzyme *AnFdc*1 from *Aspergillus niger*, reconstitution of the *apo*-*An*Fdc1 prior to molecular oxygen exposure ensures correct *in vitro* two-electron oxidative maturation (3). *An*Fdc1 thus exhibits autocatalytic oxidative maturation and avoids prFMN^.^radical formation, but the extent to which this applies across the wider UbiD family remains unclear. Indeed, *in vitro* reconstitution with prFMNH_2_, where the UbiD–prFMNH_2_ complex formation precedes oxidation, is able to support activation of a range of UbiD enzymes, albeit with varying levels of the inactive radical prFMN^.^enzyme complex as a side product (4, 5, 6, 7). The canonical *Escherichia coli* UbiD enzyme involved in ubiquinone biosynthesis, from which this enzyme family derives its name, presents an example that of a UbiD enzyme that cannot be reconstituted *in vitro*, yielding only the inactive prFMN^radical^ complex, hampering detailed biochemical studies (4). This suggests that successful oxidative maturation of this particular UbiD could require a chaperone and/or an alternative oxidant *in vivo.*

**Figure 1:**
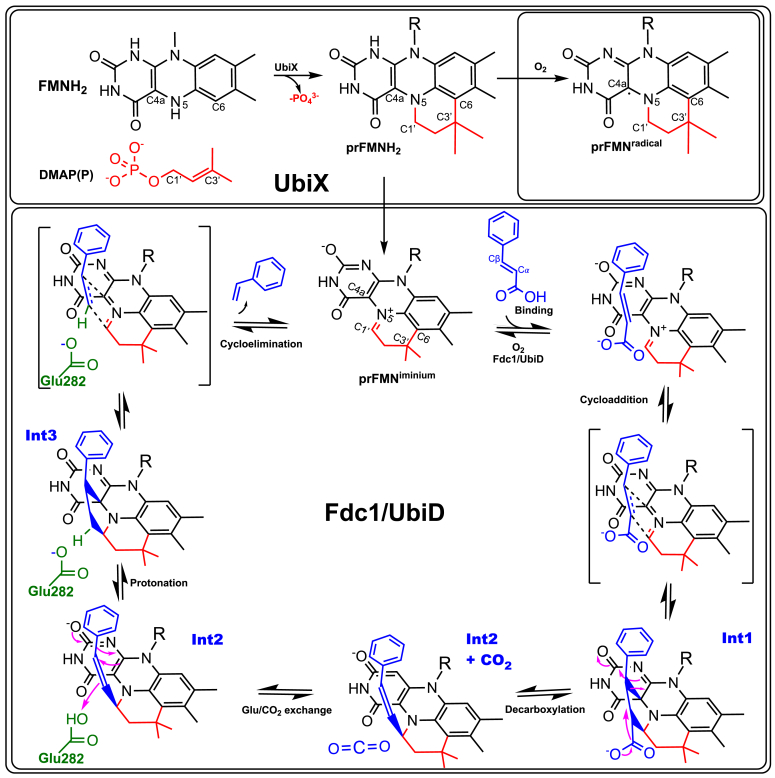
**The UbiDX system.** Chemistry of prFMNH_2_ formation by UbiX and oxidative maturation and (de)carboxylation by the UbiD family member *An*Fdc1 (**1**). prFMN, prenylated flavin.

Many UbiD homologs are found in obligate anaerobes (8, 9), whereas *E. coli* UbiD is postulated to function independent of oxygen levels (10, 11, 12), raising the possibility of other oxidative processes unconnected to molecular oxygen could take place. While no chaperone has been identified as associated with the *E. coli* UbiD, microbial genome sequences reveal there is considerable variability in genes found to be associated with UbiD. Indeed, UbiD-like genes are frequently located within operons encoding partner proteins, most frequently the UbiX flavin prenyltransferase required for cofactor synthesis (13). Aside from the obligate requirement for *ubiX* coexpression, additional non-*ubiX* genes can be present. The latter are diverse in sequence with many of unknown function. In a few cases, the corresponding role in supporting UbiD (de)carboxylation has been elucidated, such as the phosphatase subunit associated with the complex phenol phosphate carboxylase enzyme (14). In the case of the vanillic acid decarboxylase VdcC, the associated VdcD protein was shown to act as an integral part of the active VdcCD oligomer, with the isolated VdcC subunit showing no activity (7). Previous studies indicated that the small protein LpdD (lp_0272) is associated with the UbiD-type gallate decarboxylase LpdC (lp_2945) in *Lactobacillus plantarum* (15, 16).

The *lpdD* gene is cotranscribed with the UbiX-homolog *lpdB* (Fig. 2), although LpdD has been demonstrated to play little or no role in the gallic acid decarboxylation activity (16). Finally, the presence of small putative UbiD accessory genes is also reported for a range of other bacterial species (17). We show that *lpdD* heterologous expression induces a previously undetected UbiD-dependent decarboxylation activity in *E. coli.* Combined with the LpdD crystal structure and solution data, we propose that LpdD acts as a prFMNH_2_-binding chaperone, able to assist UbiD prFMN incorporation and/or oxidative maturation.

**Figure 2:**
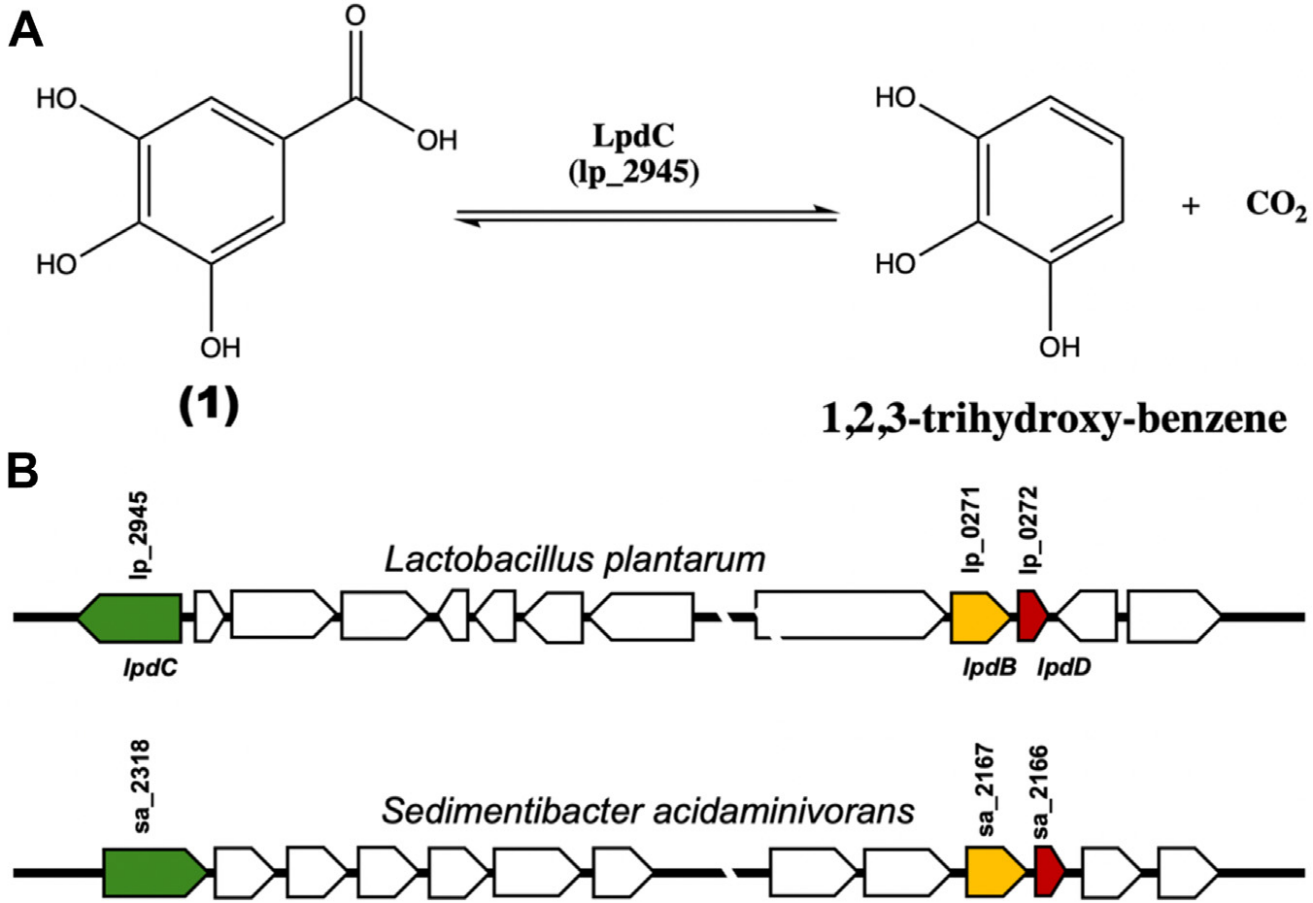
**The *Lactobacillus plantarum* gallic acid decarboxylase.***A*, LpdD catalyzes gallic acid (**1**) decarboxylation. *B*, schematic representation of the *L. plantarum* chromosome section containing the *lpdC* (lp_2945) and *lpdB/lpdD* (lp_0271/lp_0272) genes (15). LpdD subunit is represented by *red*, LpdB (Ubix homolog) shown by *yellow*, and LpdC (UbiD homolog) shown by *green*. A similar arrangement is also present in *Sedimentibacter acidaminivorans* DSM24004.

## Results

### LpdD heterologous expression, purification, and ligand-binding studies

**Figure 3:**
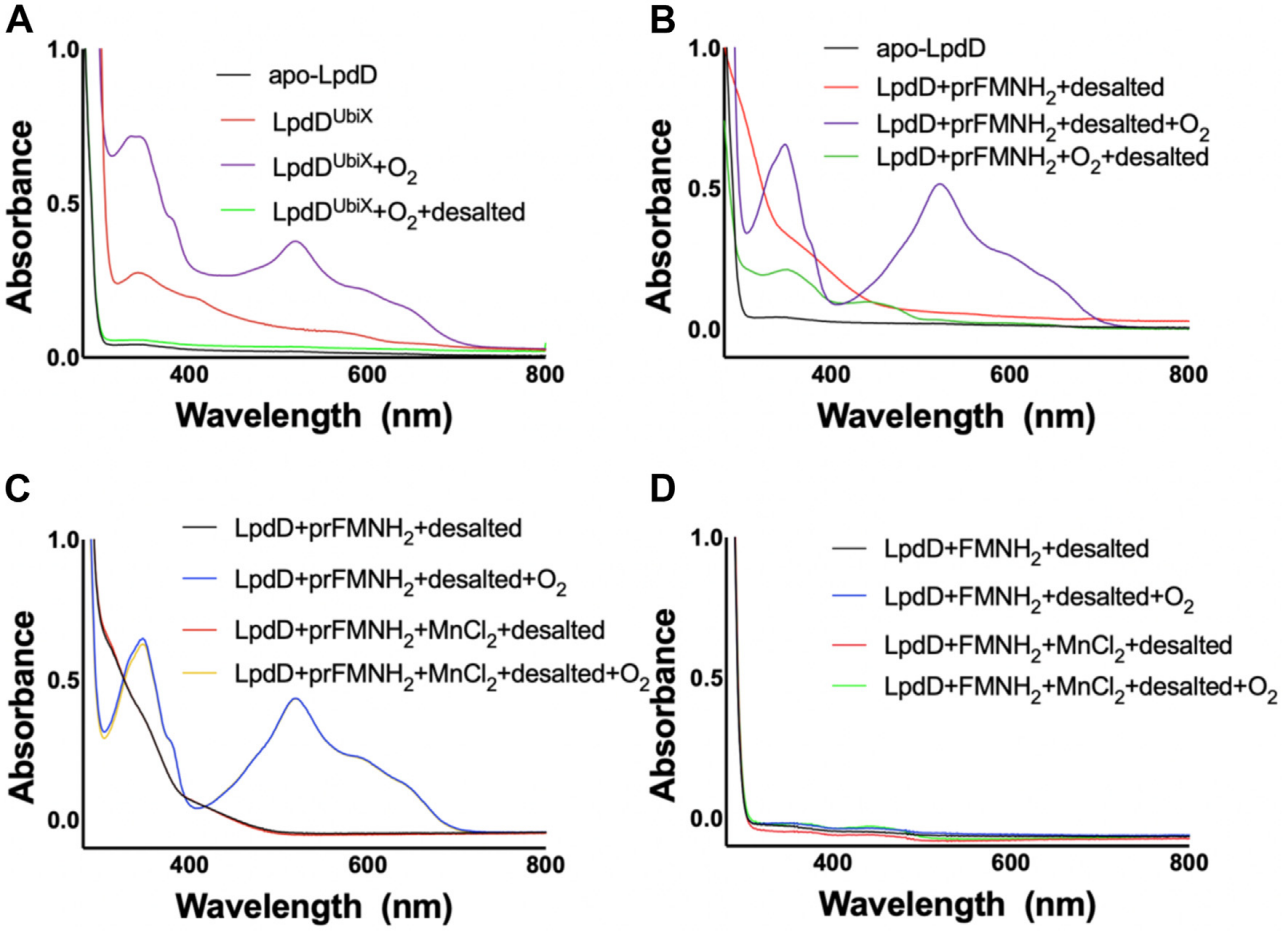
**UV–Vis spectrophotometry indicates LpdD binds prFMNH**_**2.**_*A*, in-cell prFMN binding following coexpression of LpdD with UbiX (LpdD^Ubix^) leads to prFMNH_2_ spectral features when purified under anaerobic conditions. Exposure to oxygen leads to spectral changes and a broad peak at ∼540 indicative of prFMN^radical^ formation. *B*, LpdD *in vitro* reconstitution with prFMNH_2_ leads to similar spectral features as in *A*. *C*, the presence of Mn^2+^ did not affect LpdD–prFMNH_2_ binding when reconstituted *in vitro*. *D*, no LpdD–FMNH_2_ complex could be isolated following desalting as indicated by a lack of FMN-associated spectral features. prFMN, prenylated flavin.

The *L. plantarum lpdD* was cloned with an N-terminal His tag, expressed in *E. coli*, and purified to homogeneity by a single-step purification using nickel–nitrilotriacetic acid affinity chromatography. Aerobically purified LpdD, obtained either with or without UbiX coexpression, showed no distinct spectral features. In contrast, when LpdD^UbiX^ (superscript ^UbiX^ indicating coexpression with UbiX) is purified under anaerobic conditions, the resulting pure protein is pale yellow in color. The corresponding UV–Vis spectrum (Fig. 3) resembles that observed for previously reported prFMNH_2_-bound proteins (1, 3). Upon exposure to oxygen, the purified LpdD^UbiX^ sample turns purple with the corresponding spectrum exhibiting a broad peak at ∼540 nm indicative of the formation of a semiquinone-like radical prFMN species (Fig. 3*A*).

*In vitro* reconstitution of purified *apo*-LpdD with prFMNH_2_ results in similar spectral features to those observed for the anaerobically purified LpdD^UbiX^ (Fig. 3*B*). Furthermore, a desalting step following brief exposure to oxygen readily separates the prFMN^.^radical from *apo*-LpdD, suggesting oxidized prFMN species readily disassociate from LpdD. The presence of MnCl_2_ (Mn^2+^ is required for prFMN binding in UbiD but not UbiX) in the reconstitution buffer appears to have no noticeable effect on LpdD–prFMNH_2_ binding (Fig. 3*C*). To find out if LpdD specifically binds the prenylated prFMNH_2_, as opposed to flavins more generally, we explored *apo*-LpdD reconstitution with reduced FMNH_2_ (Fig. 3*D*).

**Figure 4:**
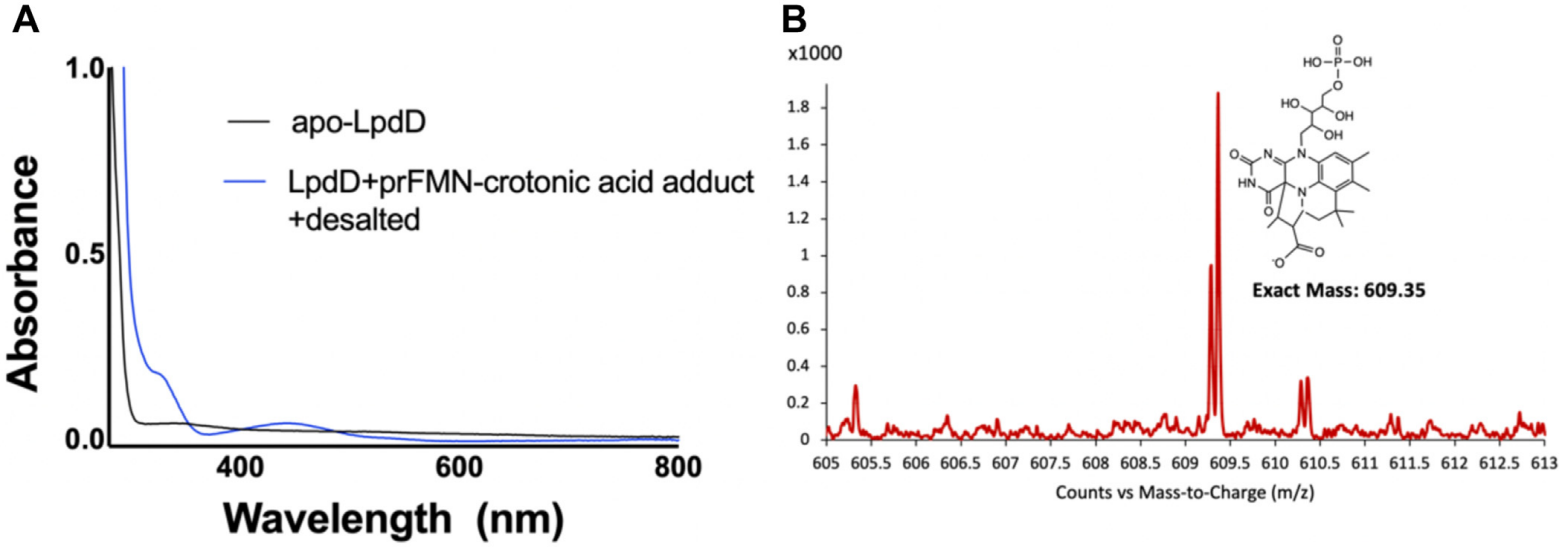
**LpdD–prFMN–crotonic acid adduct complex formation.***A*, UV–Vis spectrum of the LpdD–prFMN–crotonic acid adduct following desalting. *B*, electrospray ionization–mass spectrometry of prFMN–crotonic acid adduct (theoretical mass of M^−^ = 609.20 Da) bound to LpdD.

Neither reduced FMNH_2_ nor oxidized FMN were observed to bind to LpdD under the conditions described (Fig. 3*D*). Finally, we tested the LpdD affinity for a stable covalent prFMN–crotonic acid–derived adduct, which has prFMNH_2_-like properties but is stable under aerobic conditions and thus experimentally less challenging (18). When LpdD is reconstituted with the prFMN–crotonic acid adduct, a stable complex is formed (Fig. 4). The combined results suggest that LpdD is highly specific for reduced prFMN species.

### Reconstituted LpdD–prFMNH_2_ activates *apo*-*An*Fdc

**Figure 5:**
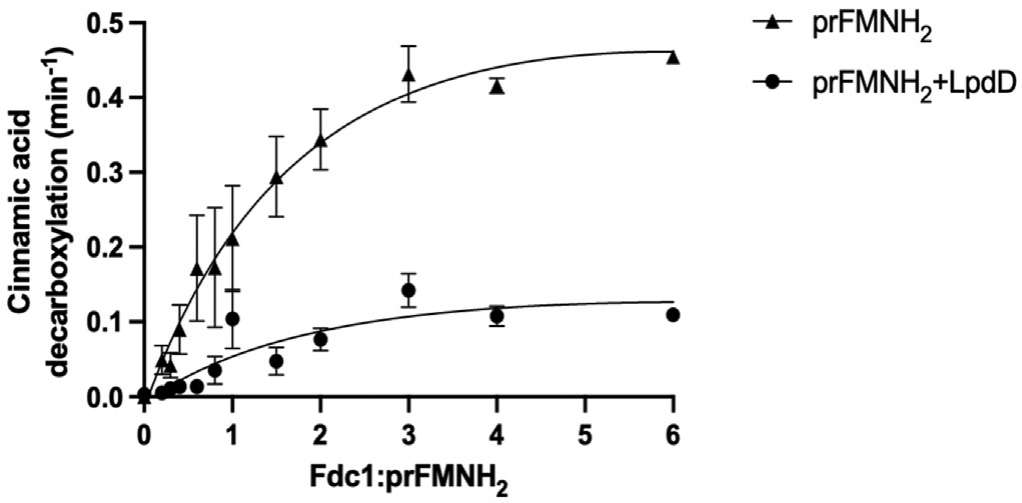
***An*Fdc1 activation by the LpdD–prFMNH**_**2**_**complex.** Plot of cinnamic acid decarboxylation activity *versus* the *An*Fdc1:prFMNH_2_ ratio in the presence or the absence of LpdD. Assays were performed in three independent replicates, data points represent mean ± SD. prFMN, prenylated flavin.

Given purified LpdD appears to specifically bind reduced prFMN species, we sought to investigate if the LpdD–prFMNH_2_ complex can activate the model UbiD enzyme *An*Fdc1 from *A. niger*. Inactive *apo*-*An*Fdc1 was added to prFMNH_2_ (either in complex with LpdD or not) in different molar ratios, and *An*Fdc1-mediated cinnamic acid decarboxylation activity was measured as an indication of active *holo*-*An*Fdc1 formation (Fig. 5). For the LpdD-containing assays, prFMNH_2_ was incubated with 10-fold excess of *apo*-LpdD prior to addition to *apo*-*An*Fdc1 to ensure full LpdD–prFMNH_2_ complex formation leaving no prFMNH_2_ free in solution. In the absence of LpdD, full *An*Fdc1 decarboxylation activity was reached with 1:3 M ratio (*An*Fdc1:prFMNH_2_). In the LpdD-containing assays, the maximum *An*Fdc1 activity following oxidative maturation was reduced to ∼30% of the previous value. This demonstrates that the LpdD–prFMNH_2_ complex can release prFMNH_2_ to activate *An*Fdc1 but acts inhibitory under the conditions used.

### LpdD expression unlocks whole-cell decarboxylation activity

**Figure 6:**
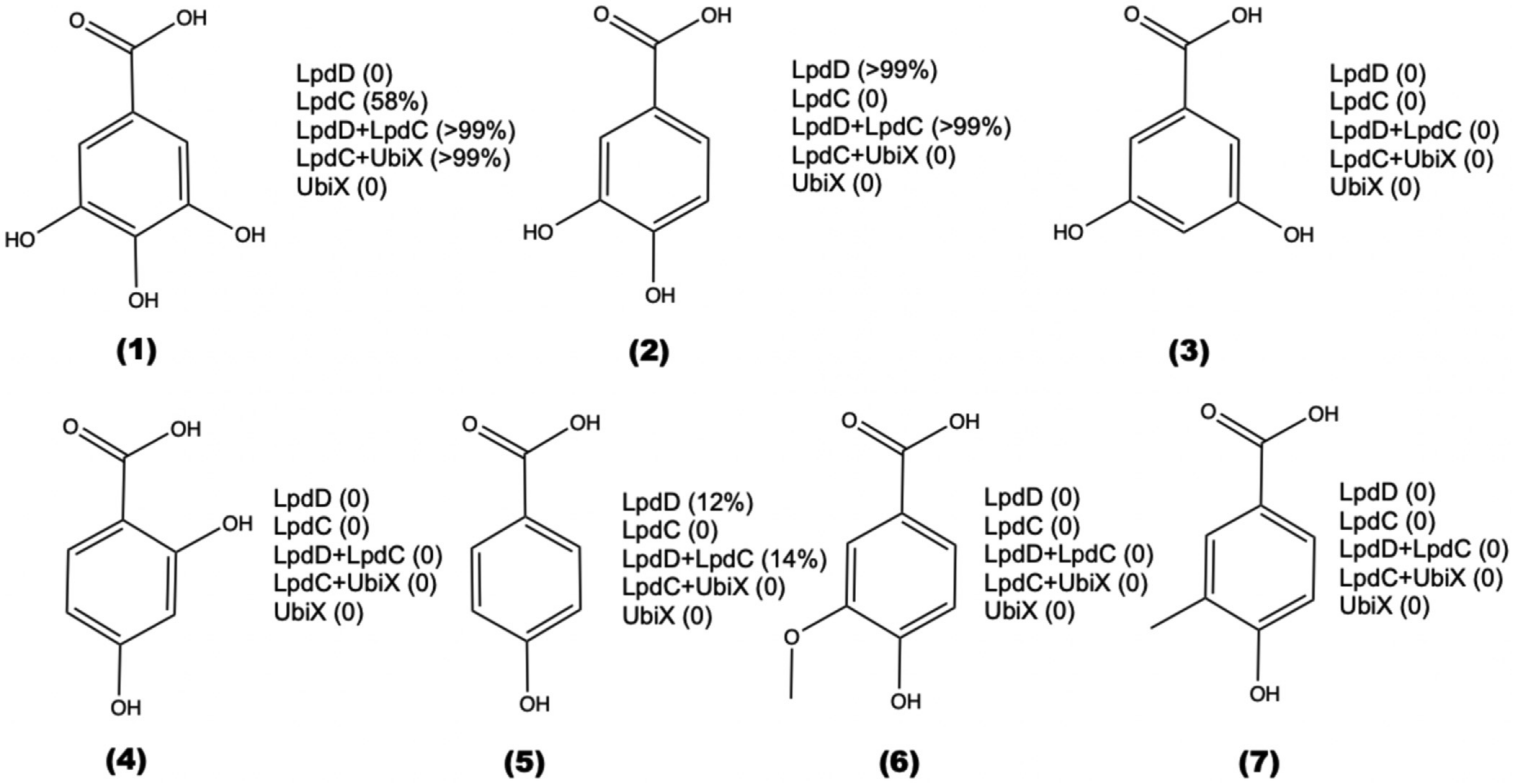
***Escherichia coli* whole-cell decarboxylation activity with a range of hydroxylated benzoic acids.** Substrate conversion levels using whole-cell lysate indicated where decarboxylation activity was observed for distinct strains. No activity was observed for compounds **3**, **4**, **6**, and **7**.

To determine the effect of LpdD expression on LpdC activity *in vivo*, we explored whole-cell activity for *E. coli* expressing either LpdD, LpdC, or both. Furthermore, we assessed the effect of UbiX coexpression. Following induction, cells expressing a diverse combination of LpdD–LpdC–UbiX were harvested and used for whole-cell transformation assays with a small library of aromatic acids. This library consisted of gallic acid (3,4,5-trihydroxy-benzoic acid; **1**), 3,4-dihydroxy-benzoic acid (**2**), 3,5-dihydroxy-benzoic acid (**3**), 2,4-dihydroxy-benzoic acid (**4**), *p*-hydroxybenzoic acid (**5**), vanillic acid (**6**), and 3-methyl-4-hydroxybenzoic acid (**7**) (Fig. 6). Cells expressing LpdC and UbiX (leading to LpdC^UbiX^ *in vivo*) demonstrated decarboxylation activity exclusively with substrate **1** as expected. In comparison, modest activity was observed when LpdC-only expressing cells (*i.e.*, no UbiX or LpdD coexpression) were assayed, likely indicating requirement for higher prFMN levels afforded by UbiX coexpression above basal levels. Interestingly, high **1** decarboxylation activity could also be observed for LpdC–LpdD-expressing cells in the absence of UbiX coexpression. This suggests that the presence of LpdD significantly enhances LpdC activation *in vivo* when under low [prFMNH_2_] conditions afforded by basal UbiX expression levels. Surprisingly, while LpdC expression was linked to **1** decarboxylation only, we also noticed significant decarboxylase activity with cells expressing either only LpdD or LpdD and UbiX against substrates **2** and **5**. No decarboxylation activity was observed in cells expressing only UbiX at high levels.

### CRISPR interference gene silencing of *ubiD* demonstrates LpdD activates *apo*-UbiD *in vivo*

The fact that LpdD-only expressing cells show decarboxylation activity toward substrate **2**, and, to a lesser extent, **5**, suggests the LpdD-dependent activation of an *E. coli* decarboxylase, in much the same manner as LpdD supports full LpdC activation under basal UbiX activity conditions. Previous studies showed that the *E. coli* genome harbors a single *ubiD* gene that plays an important role in ubiquinone biosynthesis (13), which is therefore the most likely candidate. We employed CRISPR interference (CRISPRi) gene silencing to explore whether the LpdD expression–linked aromatic acid decarboxylation phenotype is coupled to *E. coli*-UbiD expression levels. We designed two CRISPRi plasmids for *E. coli ubiD*-gene silencing, one to target the prerequisite 3 to 4 bp protospacer adjacent motif sequence (TTTV) in the promoter region, upstream of the positive 5′ end (CRP1), and another inside the 5′ end of target CDS (CRP2) (19).

**Figure 7:**
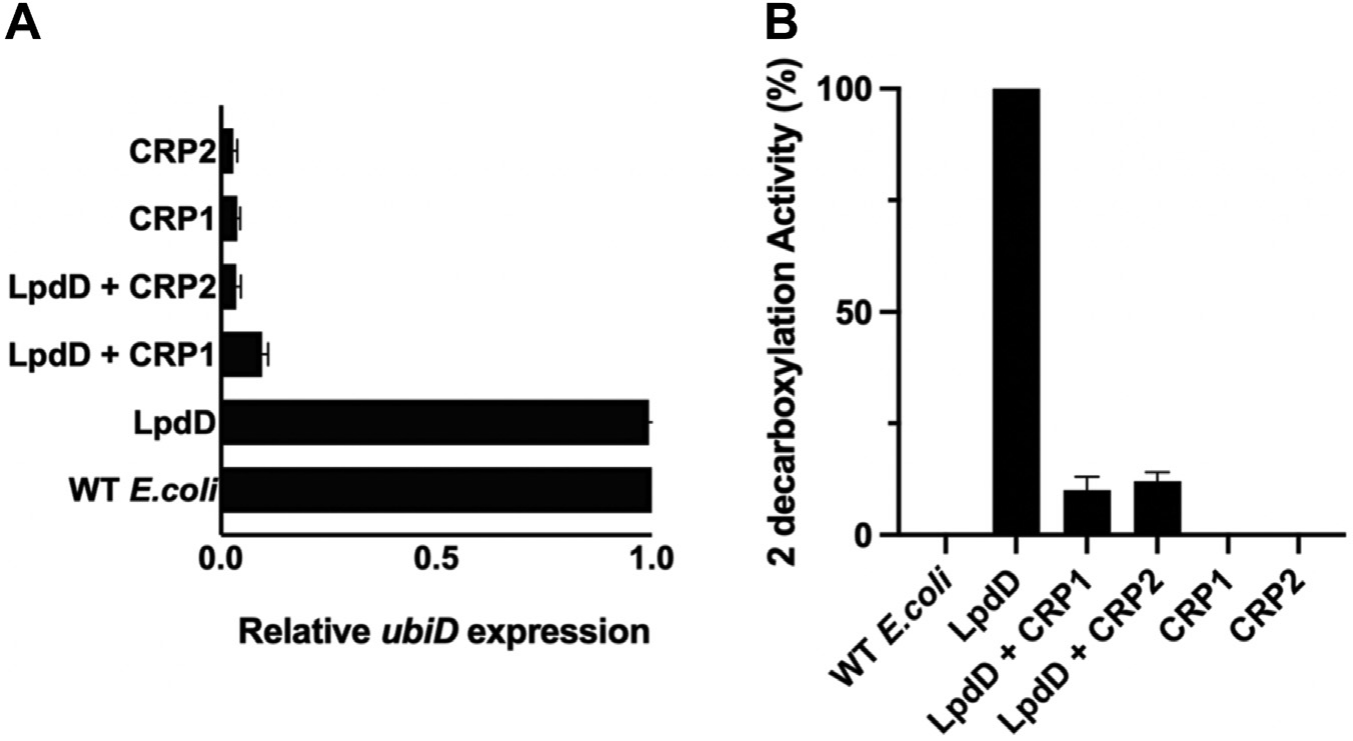
**CRISPRi repression of *ubiD* gene expression in *Escherichia coli.****A*, two different CRISPRi systems (CRP1 and CRP2) were expressed in *E. coli* cells with or without LpdD coexpression. Both CRP1 and CRP2 downregulate *ubiD* gene constitutive expression. Results are expressed relative to control *E. coli* cells with no spacer sequence plasmid. The *E. coli* housekeeping genes *idnT* and *hcaT* were used as internal control for RT–qPCR. Each sample consisted of biological triplicate, and all qPCRs were performed in technical triplicate. *B*, LpdD expression–linked **2** decarboxylation activity is reduced for CRP1 or CRP2 plasmid containing *E. coli* cells. Each sample was grown in biological triplicate, and decarboxylation reactions were performed in technical triplicate. CRISPRi, CRISPR interference; qPCR, quantitative PCR.

The CRP1/2 plasmids were transfected in *E. coli* cells, and cells were induced in early log phase and harvested during late-log phase. Quantitative RT–PCR analysis of cells expressing the CRP plasmids showed ∼90% repression of the *ubiD* gene (Fig. 7*A*). In the case of *lpdD*-expressing strains, the UbiD gene repression was ∼85% for CRP1 and ∼90% for CRP2. The decarboxylation activity for substrate **2** in LpdD-expressing cells containing either of the CRP1/2 plasmids is significantly reduced, consistent with the drastic reduction in *ubiD* expression levels (Fig. 7*B*). This suggests that LpdD assists with *apo*-UbiD activation *in vivo*, and that the resulting *holo*-UbiD is responsible for the decarboxylation activity observed in whole-cell assays.

### Crystal structure of LpdD reveals similarities with a yeast chaperone

**Figure 8:**
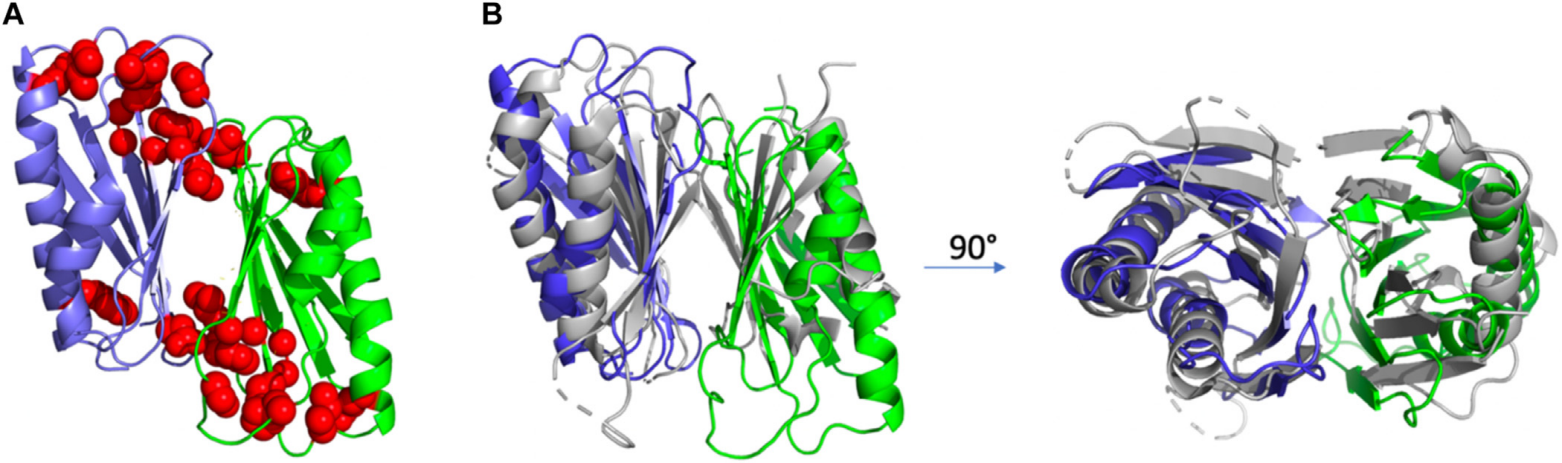
**LpdD crystal structure.***A*, the LpdD dimer with distinctly colored monomers and strictly conserved residues (Fig. S1) highlighted in *red spheres*. *B*, overlay of LpdD (similar color and orientation as *A*) with the structurally similar yeast chaperone Pba3–Pba4 (*in gray*; Protein Data Bank ID: 2Z5B) shown in two orientations.

To examine the structural basis of LpdD function, the crystal structure of *apo-*LpdD was determined using single anomalous diffraction with selenomethionine-substituted protein crystals and the final model refined against data collected on nonsubstituted protein. Crystals belong to the I222 space group with the asymmetric unit containing two LpdD monomers that superimpose with 0.11 Å rmsd. The functional dimer can be constructed from crystallographic symmetry (20). The LpdD dimer interface is composed of a β-sandwich structure formed by two six-stranded β-sheets derived from each monomer. The β-strand sandwich structure is surrounded by two alpha helices on either side of the dimer. The LpdD dimer interface is composed of approximately 27 mainly hydrophobic residues and approximately 22 Å long and 21 Å wide, burying a total surface area of 2240 Å^2^ accounting for ∼15% of the total surface (Fig. 8). The majority of the fully conserved residues (Fig. S1) cluster to one side of the monomer and involve loop regions rather than structural features.

**Figure 9:**
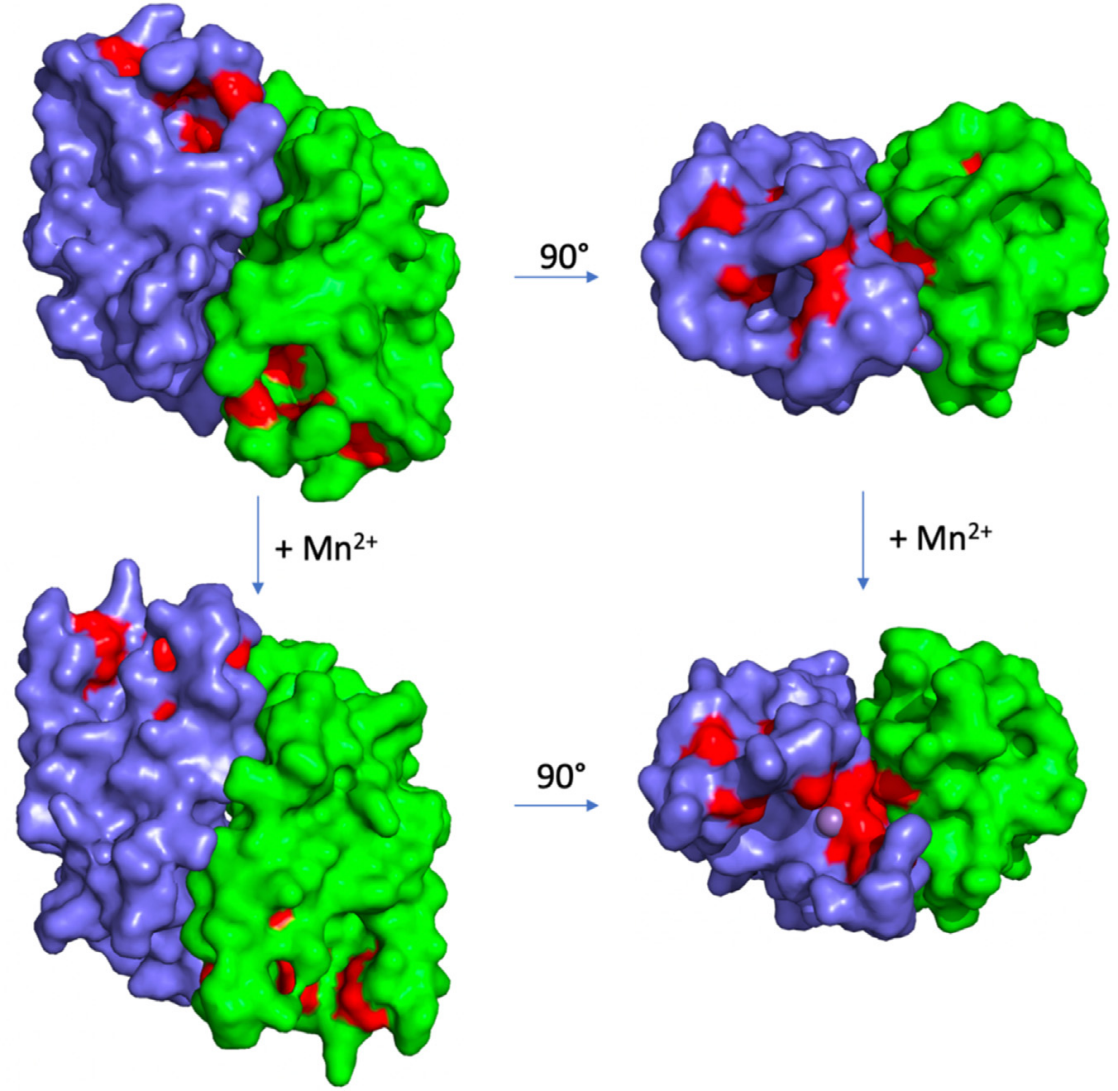
**LpdD metal binding.** The accessible surface of LpdD color coded as in Figure 8*A* shown for *apo*-LpdD and the Mn^2+^-bound LpdD in two orientations.

A structural similarity search using DALI (21) showed that LpdD monomer shares highest similarity (an Z score of 10.3) with the heterodimer yeast chaperone Pba3–Pba4 involved in 20S proteosome assembly (Protein Data Bank [PDB] ID: 2Z5B, rmsd: 2.8 Å for 105 C-αs) (22), with the LpdD dimer interface resembling the Pba3–Pba4 interaction (Fig. 8*B*). Despite multiple attempts, the crystal structure of LpdD in complex with prFMNH_2_ could not be determined, because of the difficulties associated with maintaining the strict anaerobic conditions required. However, LpdD crystals soaked with MnCl_2_ and prFMNH_2_ did reveal the presence of a metal ion in the electron density, coordinated by His35, His61, and Asp63 residues. Major conformational changes were observed when compared with the non–metal-bound *apo*-LpdD in the F53–I66 loop region, containing two of the three ligating residues (Fig. 9). This region is highly conserved (Fig. S1) despite having no obvious structural role in the *apo*-LpdD, which may indicate Mn^2+^ binding could be relevant for activity, as is observed for UbiD enzymes.

### LpdD variants affecting the metal-binding site

**Figure 10:**
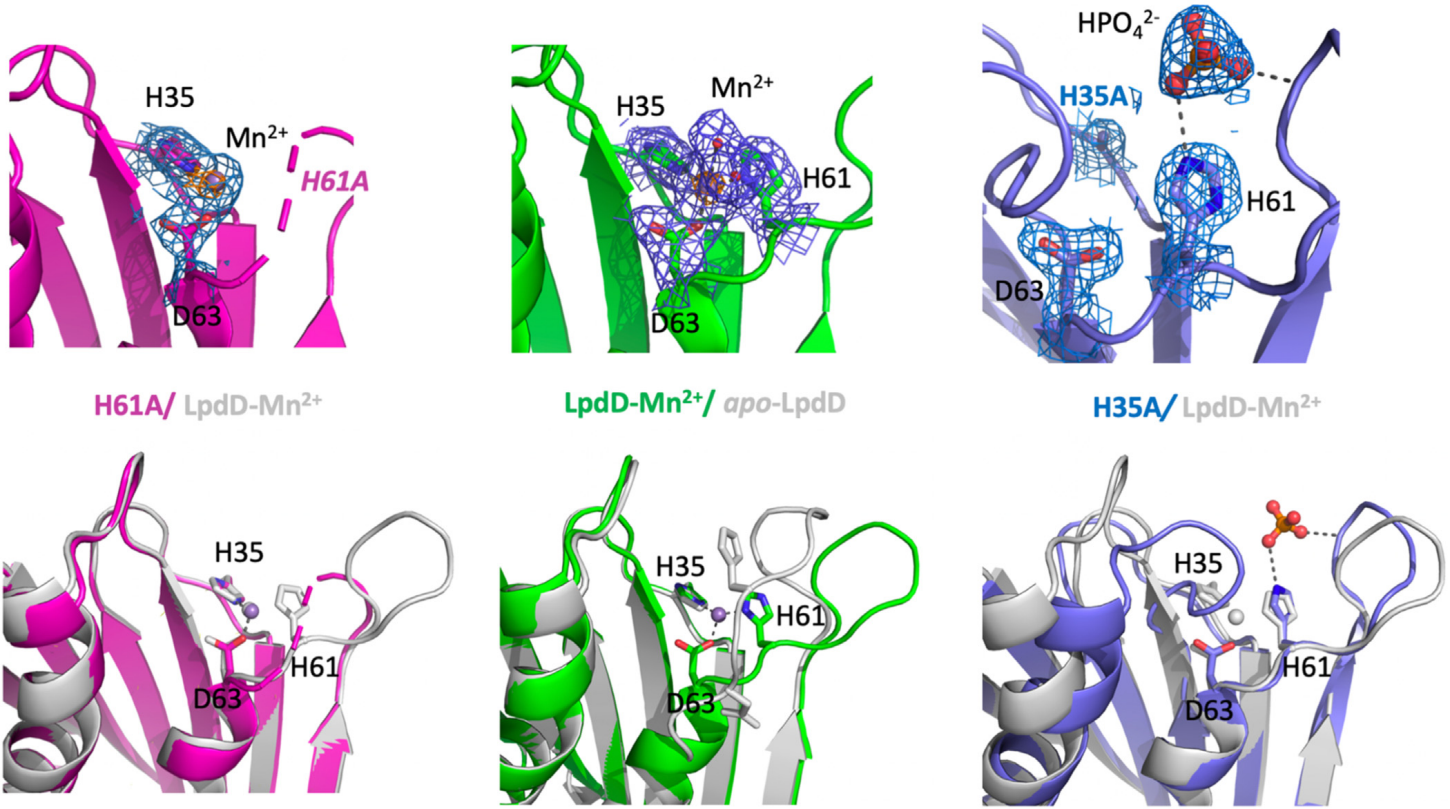
**Crystal structures of LpdD variants.** Side-by-side comparison of LpdD variants H61A (in *magenta*) and H35 (in *blue*) and the LpdD–Mn^2+^ complex (in *green*). The *top panel* shows the 2F_o_F_c_ electron density for the key metal-binding positions H35/H61/D63 contoured at 1 sigma in *blue* and 4 sigma in *orange*. The *bottom panel* shows overlays between the variants H61A (in *magenta*) and H35 (in *blue*) with the LpdD–Mn^2+^ complex (in *gray*) and an overlay between the LpdD–Mn^2+^ (in *green*) with the *apo*-LpdD structure (in *gray*).

Given both H35 and H61 are strictly conserved, we made LpdD H35A and H61A variants to test for the effect of an intact metal-binding site on prFMNH_2_ binding (Fig. 10). In the case of the H35A variant, crystals belonged to a different space group (P4_1_2_1_2) containing only one molecule in the asymmetric unit. As with the WT structure, a functional and highly similar dimer can be made by applying crystallographic symmetry (20). The H35A monomer superposed with rmsd of 0.35 Å to the Mn^2+-^bound WT LpdD, with significant conformational changes observed in loops spanning 8 to 11, 30 to 36, 54 to 62, and 89 to 95 encompassing most of the strictly conserved residues. No electron density for Mn^2+^ could be observed, but residual electron density was observed and assigned to a bound phosphate near H61. While the distinct crystallization conditions and crystal packing likely contribute to the observed changes, it is clear the presence of H35 is essential for metal binding and associated loop conformations. The H61A variant crystallized in the same space group and conditions as the WT LpdD, and superposed with the Mn^2+^-bound WT LpdD monomer (0.24 Å rmsd). The loop region spanning residues 55 to 62 was disordered for both monomers in the AU. Residual electron density was present located next to H35 and D63 that was assigned to a weakly bound Mn^2+^.

**Figure 11:**
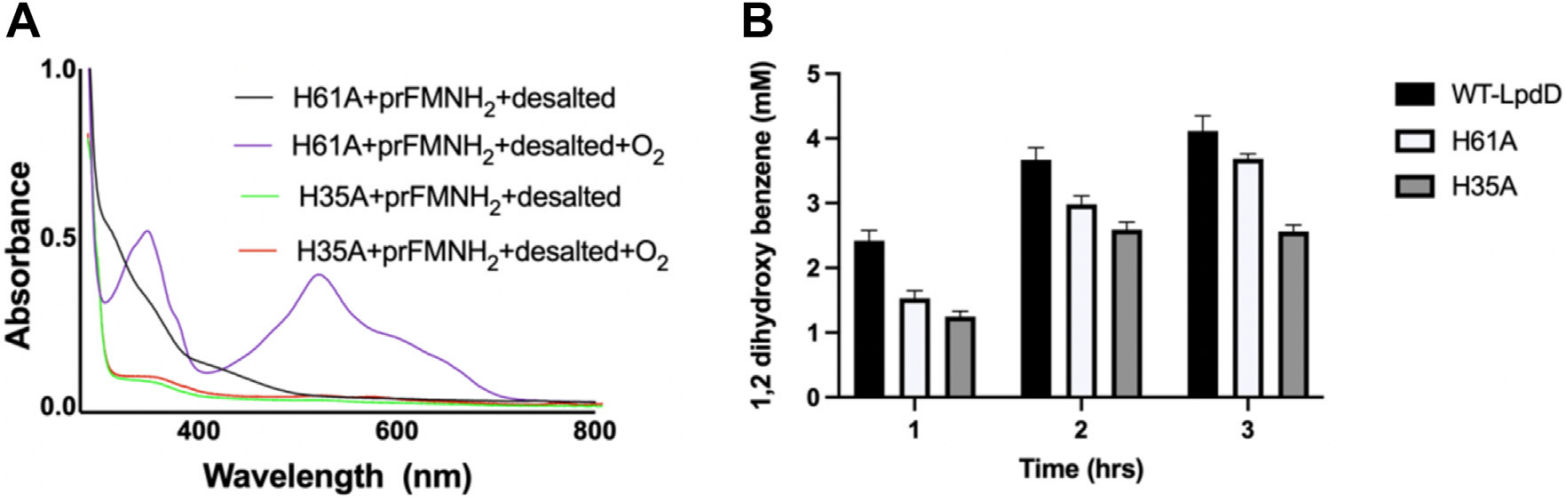
**Solution properties of the LpdD H35A and H61A variants.***A*, UV–Vis spectra of LpdD H61A and H35A variants following reconstitution with prFMNH_2._*B*, whole-cell **2** decarboxylation activity as assessed by product yield of WT-LpdD-expressing cells in comparison with cells expressing H35A and H61A variants. No significant **2** decarboxylation activity was observed in case of *Escherichia coli* cells transformed with empty plasmid. prFMN, prenylated flavin.

When tested using whole-cell decarboxylation assays, both variants displayed reduced activity when compared with WT LpdD whole-cell assays, with H35A more affected than H61A. *In vitro* reconstitution of the purified H61A variant reveals this mutation does not abolish prFMNH_2_ binding, whereas the purified H35A variant did not bind prFMNH_2_ under the conditions used (Fig. 11). This suggests that His35—and by extension metal binding—is more important for LpdD function *in vivo* compared with H61, although neither are essential under the conditions tested.

### Activation of *E. coli apo*-UbiD with LpdD

**Figure 12:**
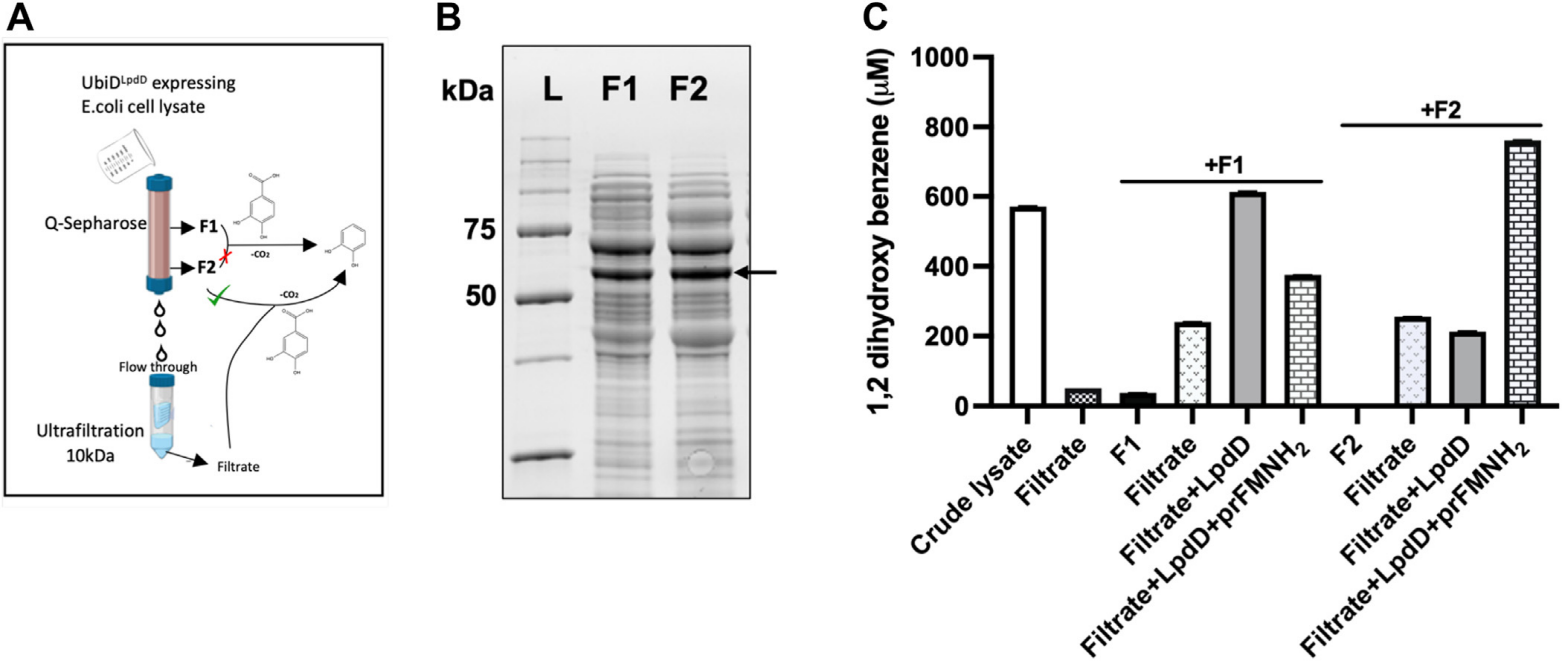
**Activation of *Escherichia coli* UbiD.***A*, schematic illustration of the experimental approach used. *B*, *E. coli* UbiD^LpdD^ was expressed and purified on Q-Sepharose column with NaCl gradient of 0 to 500 mM. Two decarboxylation assays were used to monitor the activity of fractions. Q-Sepharose flow through (FT) was passed through the 10 kDa spin concentrator and added back to the fraction F1 (100 mM) and fraction F2 (200 mm). UbiD protein presence was indicated by *black arrow* on SDS-gel. *C*, activity data of fraction F1 and fraction F2 before and after supplementing with filtrate/LpdD and or prFMNH_2_. prFMN, prenylated flavin.

Intrigued by the fact that LpdD activates *E. coli* UbiD *in vivo*, we set out to purify active UbiD coexpressed with LpdD (UbiD^LpdD^) to assess **2** decarboxylation activity *in vitro*. All purification steps and assays were carried out in anaerobic chamber (Belle Technology) operating at <1 ppm oxygen and 18 °C. During affinity purification of His-tagged UbiD^LpdD^, all activity was lost. However, **2** decarboxylation activity was observed in both cell lysate and flow through, indicating loss of activity following binding and/or elution. When using Q-Sepharose ion exchange as an alternative method for UbiD^LpdD^ purification, decarboxylation activity was observed in the flow-through and the 100 mM NaCl elution fraction (Fig. 12). Other elution fractions (ranging from 200 to 500 mM NaCl) showed no activity, although UbiD was present in the fractions clearly visualized on SDS-PAGE gel.

The flow-through was filtered through 10 kDa cutoff membrane and mixed with the 100 mM Q-Sepharose fraction F1, increasing decarboxylation activity ∼12-fold (Fig. 12*C*). This suggests that an essential low molecular weight component is lost during column wash/elution. Furthermore, upon addition of purified LpdD–prFMNH_2_, the F1 decarboxylation activity increased ∼14-fold. Similarly, the 200 mM NaCl fraction F2 decarboxylation activity could be recovered by adding flow-through filtrate or purified LpdD–prFMNH_2_ in combination with filtrate (Fig. 12*C*).

**Figure 13:**
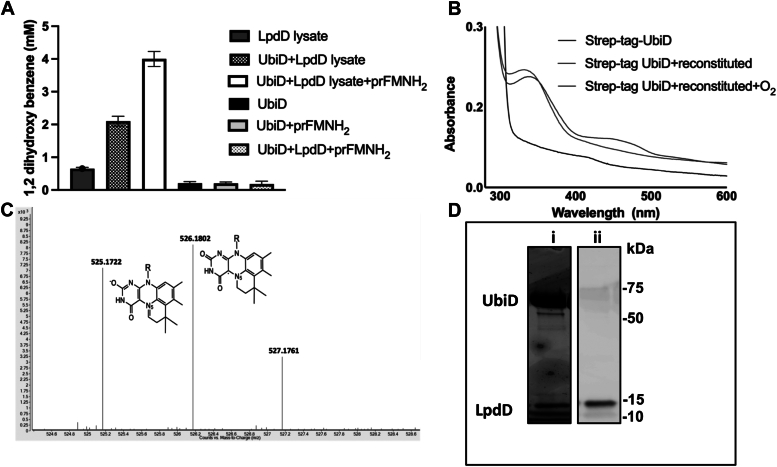
**Reconstitution of Strep-tagged UbiD.***A*, purified Strep-tagged *Escherichia coli* UbiD was supplemented with LpdD-expressing cell supernatant. Different combinations were analyzed by decarboxylation activity of two substrates. *B*, reconstitution of Strep-UbiD leads to changes in spectral features when purified under anaerobic conditions after reconstituting with LpdD-expressing cell lysate and prFMNH_2_. *C*, Electrospray ionization–mass spectrometry of activated Strep-tagged UbiD reveals the presence of prFMN^iminium^ and prFMN^.^species (525.17 and 526.18 Da, respectively). *D*, interaction studies of Strep-tagged *E. coli* UbiD and His-tagged LpdD by pull-down assays using Streptavidin beads. UbiD interacts with LpdD as detected by SDS-PAGE (i) and His-Western blot (ii) following pull down. prFMN, prenylated flavin.

Given neither F1 nor F2 fraction were pure, we repeated the activation assays using purified Strep-tagged *apo*-UbiD protein to confirm that the decarboxylation activity observed was indeed UbiD mediated. No activity was observed with the purified Strep-*apo*-UbiD alone, even when prFMNH_2_ or pure LpdD–prFMNH_2_ were added. However, activity was observed when LpdD-containing cell lysate supernatant was included in the assays (Fig. 13*A*). The further addition of prFMNH_2_ resulted in an additional twofold increase in activity. This demonstrates that the LpdD lysate can restore *apo*-UbiD activity *in vitro* and suggest that an additional soluble *E. coli* cellular component is needed, given reconstitution with purfied LpdD–prFMNH_2_ in the absence of cell lysate was unsuccessful.

Following purification of the Strep-UbiD reconstituted using LpdD-containing cell lysate, the protein spectral features reveal an absorbance feature at ∼325 nm linked to prFMN binding (Fig. 13*B*). After exposure to atmospheric oxygen, the spectral features change with appearance of a feature at ∼425 nm. These spectral features differ markedly from previously published reconstituted UbiD in the absence of LpdD (**4**), which results in a marked absorbance at ∼550 associated with the prFMN^.^species. Electrospray ionization–mass spectrometry of the corresponding reconstituted Strep-UbiD reveals the presence of catalytically relevant prFMN^iminium^ species as well as the inactive prFMN^.^radical (Fig. 13*C*) (4).

Furthermore, we aimed to investigate the interaction between UbiD and LpdD. Pull-down assays were performed by mixing Strep-tagged UbiD and His-tagged LpdD in an equimolar ratio and pulling down using streptavidin beads under anaerobic conditions. The results confirmed an interaction between LpdD and UbiD (Fig. 13*D*).

## Discussion

The involvement of chaperones in cofactor incorporation is well documented for a wide range of other systems, including the flavin complex II incorporation chaperone SdhE (23), the dedicated molybdenum insertion chaperones (24), and the ccm cytochrome *c* maturation pathway (25). However, although the key role UbiX plays in UbiD activation, that is, providing the required reduced cofactor precursor, is clearly established (2), no other prFMNH_2_-binding protein that could act as a chaperone similar to those identified in distinct cofactor-dependent systems has yet been identified. Our studies with the small protein LpdD from *L. plantarum* provide such a candidate. While LpdD is associated with the LpdC gallic acid decarboxylase, the corresponding physiological role remained unclear. We here show that LpdD coexpression enhances LpdC activity in the context of *E. coli* whole cells where only basal *ubiX* expression occurs. This suggests that the LpdD presence supports *holo*-LpdD formation and/or oxidative maturation at lower [prFMNH_2_] levels. Purified LpdD specifically binds to reduced prFMN only, despite having no sequence homology to either UbiX or UbiD, the only other prFMN-binding proteins described to date, and thus presents a new prFMN-binding fold. Structure elucidation reveals LpdD is similar in structure to a eukaryotic chaperone involved in 20S proteosome assembly, with a highly conserved region exposed at the protein surface. The latter includes a metal-binding site with possible relevance to function, with the conserved H35–H61–D63 providing a Mn^2+^-binding site. The coordination pattern allows a fourth nonprotein ligand to bind, possibly the prFMN phosphate when in complex with the cofactor, thus resembling the UbiD Mn^2+^-assisted prFMN binding. Unfortunately, the details of prFMNH_2_ binding by LpdD could not be resolved because of the prFMNH_2_ oxygen-sensitive nature and the inability of LpdD to bind the oxidized forms. Docking of prFMNH_2_ to the available LpdD structures reported here did not yield a plausible model that could account for the unusual specificity for the reduced form and lack of affinity for non-prFMN. Given the LpdD conserved surface–exposed region largely consists of loop regions that are shown to alter conformation in response to either metal binding or single-site mutations, we suggest that the LpdD–prFMNH_2_ complex likely has a distinct conformation through induced fit binding, hindering accurate modeling of the complex on the basis of the non–cofactor-bound structures. While mutagenesis of either H35 or H61 affects metal binding and, in the case of H35, prFMNH_2_ binding *in vitro*, both variants retain residual activity when assayed in *E. coli* whole-cell context. Furthermore, WT LpdD prFMNH_2_ binding occurs in the absence of Mn^2+^, which suggests metal binding is not essential for activity. Therefore, while LpdD clearly supports enhanced LpdC activity in the presence of basal UbiX levels, the exact mode of action remains unclear but is likely to include selective prFMNH_2_ binding.

**Figure 14:**
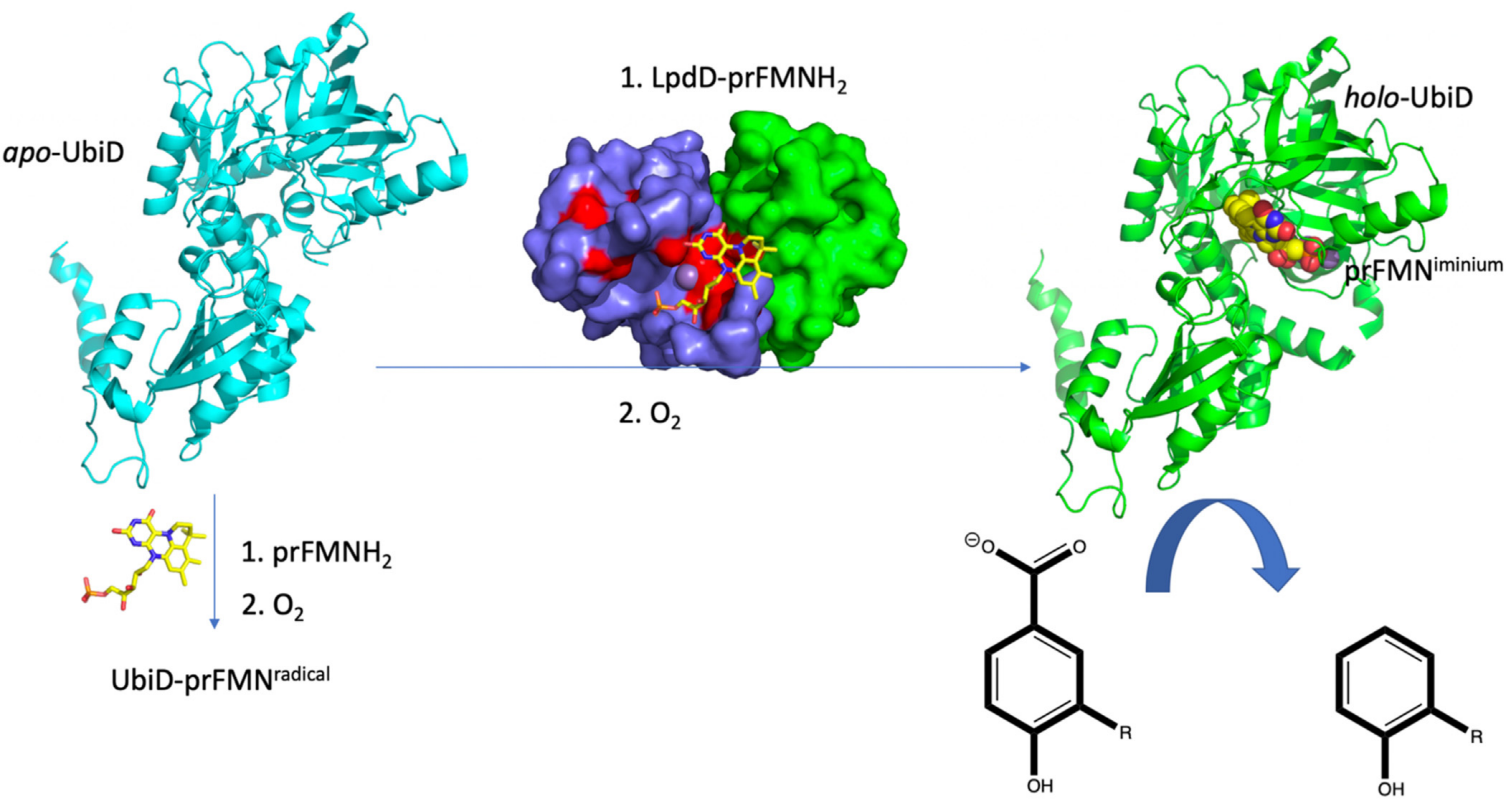
***In vivo* LpdD-mediated activation of UbiD.** Schematic proposal of activation of *Escherichia coli* UbiD by the LpdD–prFMNH_2_ complex *in vivo* (requiring an as yet unidentified additional cellular component) leading to decarboxylation activity. The nature of R is restricted to either -OH or -H. The exact binding mode of the prFMNH_2_ to LpdD could not be determined. In the absence of LpdD, only the inactive UbiD–prFMN^radical^ complex can be obtained (4). prFMN, prenylated flavin.

Surprisingly, LpdD expression in *E. coli* yielded **2** decarboxylation activity not otherwise readily detected. While this activity is typical of a UbiD class of enzyme, such as AroY (6) or VdcCD (7), the *E. coli* genome only contains a single UbiD associated with Q biosynthesis (11). It has long been proposed that UbiD catalyzes the decarboxylation of a prenylated precursor molecule, following prenylation of the soluble aromatic acid precursor by the membrane-bound UbiA. How the soluble UbiD accesses the essentially insoluble prenylated substrate has not been elucidated, although downstream processes have been shown to include carrier-type proteins (10). Furthermore, structure elucidation of the UbiD–prFMN complex did not reveal the presence of a hydrophobic region that could accommodate the prenyl chain (4). In the absence of active UbiD, biochemical studies to determine substrate scope have not been possible. We here show that UbiD is activated when in the presence of LpdD–prFMNH_2_ and an unidentified cellular component (Fig. 14). This activity is limited to **2**—and to a lesser extent **5**—decarboxylation, with no activity detected for other soluble aromatic acids that resemble the proposed prenylated substrate head group such as **6** or **7**. Unfortunately, complete purification of the active *holo*-UbiD following reconstitution using LpdD–prFMNH_2_ extract has proven challenging, either because of oxygen sensitivity or loss of a weakly bound component essential for activity. Thus, robust quantitative assessment of activity with a range of substrates is as yet out of scope. However, the observed activity profile suggests the possibility that UbiD-mediated decarboxylation could precede the UbiA-dependent prenylation step.

In contrast to UbiX, LpdD is only rarely present alongside UbiD, which might suggest that few UbiD enzymes need a prFMNH_2_-binding chaperone. However, no LpdD homologs can be found in *E. coli*, suggesting another as yet unidentified prFMNH_2_-binding chaperone occurs in this microbe. Hence, while a single highly conserved flavin prenyltransferase UbiX is found associated with UbiD enzymes, considerable diversity might be expected in the nature of the UbiD maturation pathway, ranging from robust autocatalytic to chaperone-mediated processes. The biotechnological potential of the UbiD enzyme family has recently been demonstrated through reports of *in vivo* butadiene (26) and isobutene (18) production, supported by insights into prFMN production (27, 28).

While autocatalytic cofactor maturation in the fungal *An*Fdc1 presents an efficient route to *holo*-enzyme formation, requiring no additional components apart from *apo*-enzyme and cofactor precursor (29), it requires the enzyme active site to be able to catalyze both the maturation process as well as the substrate conversion, and thus likely places constraints on the latter process. Such constraints might place limits on substrate and/or product scope and could be circumvented where required by recruitment of a chaperone. In the case of the widespread UbiD enzyme family, considerable variability in terms of oxygen sensitivity, substrate specificity, and oligomeric structure has emerged from the relatively few members that have been studied in detail (4, 5, 6, 7, 10, 12, 18, 30). This extends to the levels of activation achieved using *in vitro* reconstitution, which vary from highly efficient in case of *An*Fdc1 to entirely unsuccessful in the case of *E. coli* UbiD (4). The exact mode of action for the LpdD prFMN chaperone remains unclear, requiring further characterization of the highly oxygen-sensitive LpdD–prFMNH_2_ complex. Nevertheless, as the first example of a protein assisting the activation of a UbiD enzyme, it suggests that coexpression with both flavin prenyl transferase UbiX and a prFMN chaperone such as LpdD could be required to unlock the full (de)carboxylation scope of the UbiD-enzyme family.

## Experimental procedures

### Cloning and mutagenesis

The *E. coli* codon-optimized *lpdD* (lp_0272) and *lpdC* (lp_2945) genes from *L. plantarum* were synthesized by GeneArt (ThermoFisher). PCR was performed with Phusion polymerase (NEB). Genes were subcloned into pET30a and pET28a expression vectors with a C-terminal and N-terminal hexahistidine tag, respectively, using In-Fusion ligation-independent cloning kit (Clontech). DNA construct sequences were confirmed (Eurofins Genomics sequencing), and the purified plasmid transformed into *E. coli* BL21(DE3) for protein expression (NEB). In order to coexpress LpdC decarboxylase with UbiX, BL21(DE3) cells were cotransformed with *Pseudomonas aeruginosa ubiX* (pCDF-ubiX) together with the decarboxylase plasmid. Site-specific changes in WT constructs were introduced by the Q5 site–directed mutagenesis (NEB) method, and once the presence of the desired mutation was confirmed by DNA sequencing, the plasmid was transformed into *E. coli* BL21(DE3). LpdD was also cloned in pET21a expression vector for the coexpression of LpdD with other proteins (LpdC, UbiX) for use in whole-cell assays.

### Protein expression and purification of hexahistidine-tagged proteins

Proteins were overexpressed in *E. coli* BL21(DE3) cells grown at 37 °C either in Terrific broth media with induction by 0.25 mM IPTG overnight at 20 °C or in Terrific broth autoinduction media (Formedium) at 24 °C for 48 h. For anaerobic purification, all steps were carried out in an anaerobic chamber (Belle Technology) operating at <1 ppm oxygen and 18 °C. Cells were resuspended in anaerobic 50 mM Tris (pH 7.5), 200 mM NaCl, and 10% (v/v) glycerol (buffer A), and DNase, RNase, and SigmaFast EDTA-free protease inhibitor cocktails were added (Sigma). Cells were lysed by passage through a French pressure cell at 17.5 kpsi with sample and collection bottles under a constant flow of nitrogen gas. Cell lysates were clarified by ultracentrifuge at 185,000*g* for 1 h at 4 °C. The supernatant was applied to a 5 ml nickel–nitrilotriacetic acid agarose gravity flow column (Qiagen) in the anaerobic chamber. The resin was washed with buffer A followed by additional wash steps with buffer A containing 10 and 30 mM imidazole, respectively. Protein was eluted using buffer A containing 200 mM imidazole. Samples from the lysate, wash, and elution fractions were analyzed by 4 to 20% SDS-PAGE to determine fractions containing the protein of interest, and imidazole was removed from these fractions using a PD10 desalting resin (Bio-Rad). Purified protein was flash frozen in small aliquots (less than 50 μl) and stored in liquid nitrogen for further experimentation. Selenomethionine-labeled protein was produced in BL21(DE3) grown in media designed to inhibit prototrophic methionine production (31). Purification of SeMet-labeled *apo*-LpdD was performed as described previously in aerobic conditions.

### LpdD reconstitution with reduced prFMNH_2_ and reduced FMNH_2_

A typical prFMN production reaction, containing 1 mM FMN, 2 mM DMAP (the dimethylallylphosphate prenyl donor), 5 mM NADH, 50 μM Fre reductase (32), and 50 μM UbiX in 50 mM Tris (pH 7.5), 200 mM NaCl, was incubated at room temperature for a minimum of 4 h in an anaerobic glove box operating under 100% N_2_ (Belle Technology). The reaction mixture was filtered through a 10 kDa molecular weight cutoff spin concentrator (Vivaspin) to remove UbiX and Fre reductase proteins from the reaction mixture. Filtered prFMNH_2_ cofactor mix was added to apo-LpdD protein in the presence of 1 mM MnCl_2_ in a molar ratio of 2:1 and incubated for 10 min. Excess prFMNH_2_ cofactor was removed by passage through a PD10 desalting column (GE Healthcare) equilibrated in buffer A plus 1 mM MnCl_2_. The UbiX used in the production of prFMN is *P. aeruginosa* UbiX (PA4019) heterologously expressed in *E. coli* (2, 3). Reduced FMNH_2_ was prepared by dissolving FMN in the glove box in anaerobic buffer A and adding an equimolar concentration of anaerobic sodium dithionite. FMNH_2_ was mixed with purified LpdD in 2:1 M ratio and incubated at room temperature for 10 min. Incubation mixture was buffer exchanged, and spectra were normalized to give a comparison between proteins before and after reconstitution. All the spectral studies were recorded by UV–Vis spectroscopy using a Cary 50 Bio spectrophotometer (Varian). Protein concentrations were estimated from the absorbance at 280 nm absorption peak using extinction coefficients of LpdD (ε_280_ = 12,950 M^−1^ cm^−1^). Extinction coefficients were calculated from the primary amino acid sequences using the ExPASy ProtParam proteomics server (33). All spectra have been normalized for protein content.

### *In vitro* prFMN–crotonic acid adduct synthesis and LpdD reconstitution

An Fdc variant from *Trichoderma atroviride* was purified, and the prFMN–crotonic acid adduct was formed as described previously (18). The prFMN adduct was extracted and freeze dried as described (1, 34, 35). Freeze-dried prFMN–crotonic acid adduct (18) was mixed with purified *apo*-LpdD in the glove box and incubated for 10 min. LpdD prFMN–crotonic acid adduct mixture was buffer exchanged on a PD10 desalting column equilibrated in buffer A. Spectra were recorded before and after reconstitution and normalized for protein content. Mass spectrometry data were collected as described (18).

### *Apo*-*An*Fdc purification, reconstitution, and decarboxylation assays

*Apo*-*An*Fdc was purified as described previously (36) and, for activation assays, it was mixed with LpdD and prFMNH_2_ in different molar ratios. Activation of *apo*-Fdc was measured using 2 mM cinnamic acid in 50 mm KPi (pH 6.5), 150 mM KCl, and 1 mM MnCl_2_. All assays were analyzed by HPLC to determine styrene production. Reactions were quenched by the addition of an equal volume of acetonitrile containing 0.1% TFA and centrifuged at 16,100*g* to remove the precipitate. Sample analysis was performed using a 1260 Infinity Series HPLC (Agilent). The stationary phase was a Kinetex 5 μm C18 100 Å column, 250 × 4.6 mm. The mobile phase was acetonitrile:H_2_O (70:30) (v/v) with 0.1% TFA at a flow rate of 1 ml min^−1^. For positive control, purified *apo*-*An*Fdc was reconstituted with prFMN in the anaerobic glove box as described previously (36).

### Interaction studies of LpdD and *E. coli* UbiD

Purified LpdD- and Strep-tagged *E. coli* UbiD were mixed under anaerobic conditions in equimolar concentration and incubated on ice for 30 min. A sample (100 μl) was injected on a Superdex 200 analytical column (GE Healthcare), and eluted samples were collected and analyzed by 4 to 20% SDS-PAGE gel and His-Western blot.

### Whole-cell decarboxylation assays

All the constructs used for the whole-cell assays were transformed in *E. coli* BL21(DE3) cells. For a negative control, pET28a was transformed in *E. coli* BL21(DE3) cells. All the constructs were either expressed with autoinduction media or IPTG induction (0.2 mM). Cells were resuspended in the assay buffer (50 mM KPi [pH 6.5] and 150 mM NaCl) and incubated with 5 mM substrate with a final cell absorbance of 30 at 600 nm. Samples (typically volume = 500 μl) were taken at different time points. Reactions were quenched by the addition of an equal volume of acetonitrile containing 0.1% TFA and centrifuged at 16,100*g* to remove the precipitate. Sample analysis was performed using a 1260 Infinity Series HPLC (Agilent). The stationary phase was a Kinetex 5 μm C18 100 Å column, 250 × 4.6 mm. The mobile phase was 90:10 (v/v) acetonitrile:H_2_O for gallic acid and 50:50, with 0.1% TFA, for all other substrates at a flow rate of 1 ml min^−1^.

### *E. coli* UbiD gene silencing using CRISPRi

The *ubiD* gene in *E. coli* was silenced using the CRISPRi method (19). In brief, CRISPRi plasmids (pBbB2c-ddcpf1-rfp) were cloned with a spacer sequence: one sequence (CRP1) targeting the 5′ end of the *ubiD* gene promoter sequence and other sequence (CRP2) within the beginning of the *ubiD* gene in the *E. coli* genome. *E. coli* cells were transformed with the CRISPRi plasmids either alone or cotransformed with the pET28a-*lpdD* vector.

### Quantitative detection of *E. coli* UbiD gene regulation by CRISPRi

All the CRISPRi plasmid (CRP1 and CRP2) variants with and without pET28a-*lpdD* plasmid were transformed freshly into *E. coli* BL21(DE3) cells, and triplicate colonies were grown overnight in LB media (containing antibiotics as appropriate). Overnight grown cultures were used to inoculate 5 ml fresh media at a 1/50 dilution into 50 ml conical tubes. Cultures were grown to early log phase with an absorbance of 0.2 to 0.4 at 600 nm at 30 °C with shaking at 180 rpm. At this stage, cells were induced with 10 mM l-arabinose and 0.1 mM IPTG. All cultures were further incubated at 30 °C with shaking at 180 rpm. When the absorbance reached 1 to 1.2 (3–4 h) at 600 nm, cultures were centrifuged at 14,000*g* for 5 min, the supernatant was discarded, and pellet snap frozen in liquid nitrogen. RNA was extracted using the TRIzol Plus RNA purification kit according to the manufacturer’s instructions (Invitrogen). Quality and quantity of total RNA was determined using a Nanodrop spectrophotometer. Total RNA integrity was assessed using the Agilent 2100 Bioanalyzer with Prokaryote RNA 6000 Nano-chip. Only RNA sample with RNA integrity number above 7 were used for the downstream processing (37). Complementary DNA (cDNA) synthesis of isolated total RNA (0.5 μg) was carried out using the Superscript IV First-strand synthesis system according to the manufacturer’s instructions (Invitrogen). Any traces of genomic DNA were treated with DNase (RNase free) on column. Primers for quantitative PCR (qPCR) were designed using IDT PrimerQuest tool (Tables S1 and S2). qPCR was performed using SsoAdvanced Universal SYBR Green Supermix (Bio-Rad) in 20 μl reactions in CFX Connect Real-Time PCR system (Bio-Rad Laboratories Ltd) following the manufacturer’s instructions. cDNA was diluted 1 in 5. Each qPCR mix contained 2 μl cDNA with a final concentration of 1× Syber mix (ThermoFisher) and 300 nM forward and reverse primers. qPCR cycle parameters used were as follows: initial denaturation at 98 °C and 30 s annealing and extension at 57 °C. Amplification specificity was confirmed by melting curve analysis following the qPCR. Quantification cycle (*C*q) was calculated using the CFX Manager, version 3.0 software (Bio-Rad Laboratories Ltd). Expression of the *ubiD* gene was normalized to reference genes hcaT and idnT (Tables S1 and S2). Primer efficiency data are shown in Fig. S2.

### Crystallization, data collection, and structure determination

Both N- and C-terminal His-tagged LpdD protein was freshly purified and concentrated to *ca* 15 mg/ml before crystallization trials. Initial crystallization screening was performed with a mosquito nanodispenser (TTP LabTech) in sitting drop vapor diffusion plates using commercially available crystallization screens (PACT, JCSG, SG1, Morpheus I & II) (Molecular Dimensions). Needle and plate-like LpdD crystals appeared overnight in several conditions across the screens at 20 °C for both N- and C-terminal His-tagged LpdD. The best diffracting crystals of LpdD appeared in the E6 condition of the SG1 screen (1.6 M magnesium sulfate heptahydrate and 0.1 M Mes [pH 6.5]) from N-terminal-tagged LpdD protein. Selenomethionine-labeled crystals were also grown in same condition. Crystals were cryoprotected in reservoir solution supplemented with 10% PEG_200_ and flash frozen in liquid nitrogen. High redundancy data from selenomethionine-labeled LpdD crystal were collected at the selenium K absorption edge (wavelength = 0.9795 Å) at Diamond Light Source. The structure was solved using single anomalous diffraction phasing method using the scaled and merged data from automatic processing pipeline and further processed through AutoSol method (38) implemented in the Phenix (https://phenix-online.org/) Software package (39). The initial selenium derivative structure was used for the molecular replacement with data collected from native crystals, which diffracted up to 2.0 Å. Molecular replacement solution was found by Phaser (40), further refinement was carried out using Phenix.refine (41) and manual rebuilding in COOT (42, 43). All the data collection and refinement statistics of all the structures reported here is described in Table S3.

## Data availability

The atomic coordinate and structure factor (PDB codes: 8PO5, 8PZO, 8P4W, and 8PZH) have been deposited to the PDB (http://www.pdb.org).

## Supporting information

This article contains supporting information (44).

## Conflict of interest

The authors declare that they have no conflict of interest with the contents of this article.
