# Supplementary material for: The prFMNH2-binding chaperone LpdD assists UbiD decarboxylase activation
Source: J Biol Chem. 2024 Jan 13;300(2):105653. doi: 10.1016/j.jbc.2024.105653 (PMC10865409; doi:10.1016/j.jbc.2024.105653)
Supplement: Supplementary data [file mmc1.docx]

| 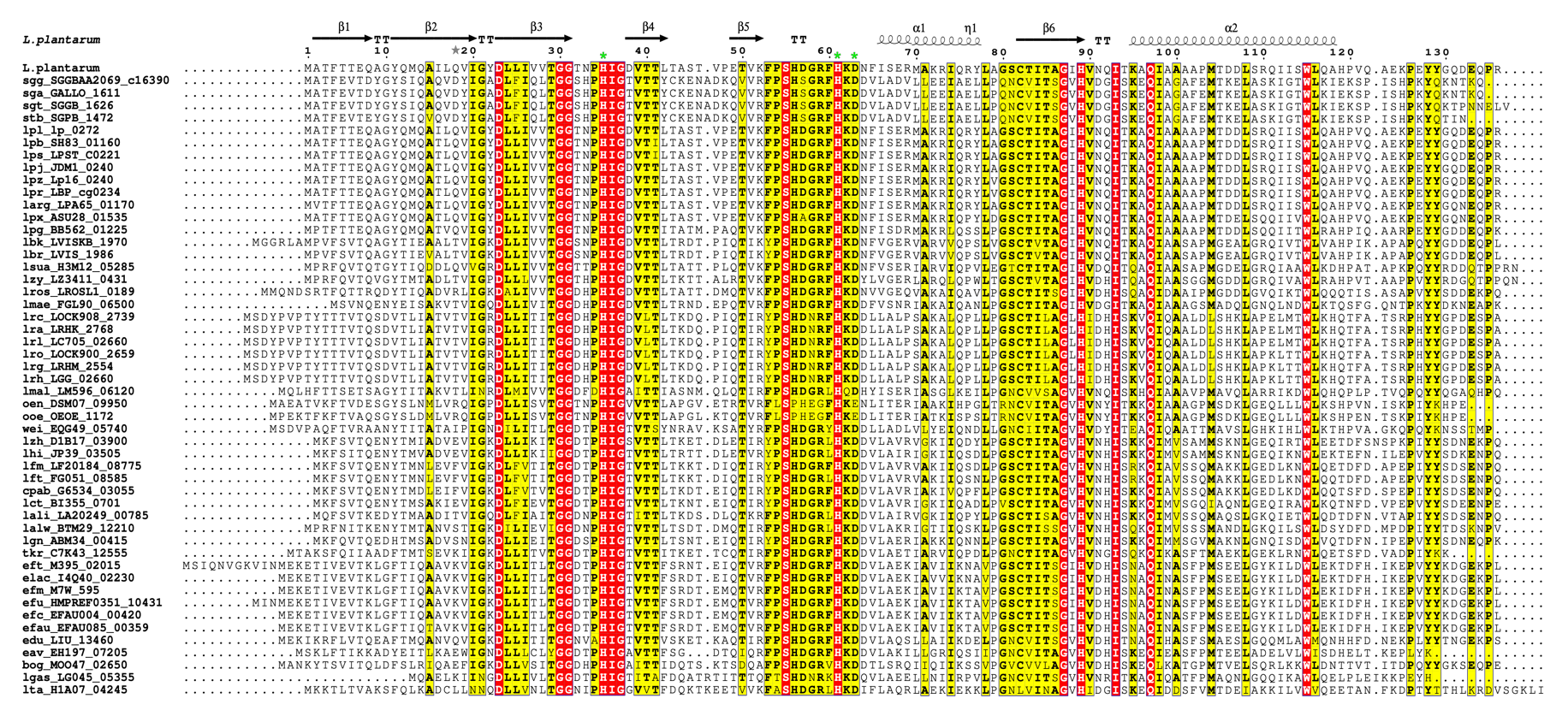 |
| --- |
| **Supplementary Figure 1**. **Alignment of LpdD sequences.** Strictly conserved residues are highlighted in red. Sequence number and secondary structure features (derived from the crystal structure presented here) are annotated according to the *L. plantarum* LpdD. Putative metal binding residues are indicated by a green * (44). |

**Supplementary figures and tables**

**Supplementary Table 1**. Summary of primer sequences for RT-qPCR.

|  |  | Primer Sequence (5’-3’) |
| --- | --- | --- |
| **UbiD** | Forward | GCTGGATGCCACGAATAAATG |
|  | Reverse | CATCCCAGATGGCGTCAATA |
| **hCTA** | Forward | CCGTTTACAGGCGGTTTACT |
|  | Reverse | CCGTGGCCCAGATATTGATAC |
| **idnT** | Forward | GTGCGCCTCTTCTTTGAATTT |
|  | Reverse | TCGATGGTGCGTCCATTAC |

**Supplementary Table 2**. Summary of RT-qPCR primer efficiency data.

|  | **UbiD** | **idnT** | **hCTA** |
| --- | --- | --- | --- |
| **R2** | 0.99 | 0.99 | 0.99 |
| **Slope** | -3.52 | -3.46 | -3.58 |
| **Amplification factor** | 1.92 | 1.95 | 1.9 |
| **Efficiency** | 92.35 | 94.54 | 90.25 |

| 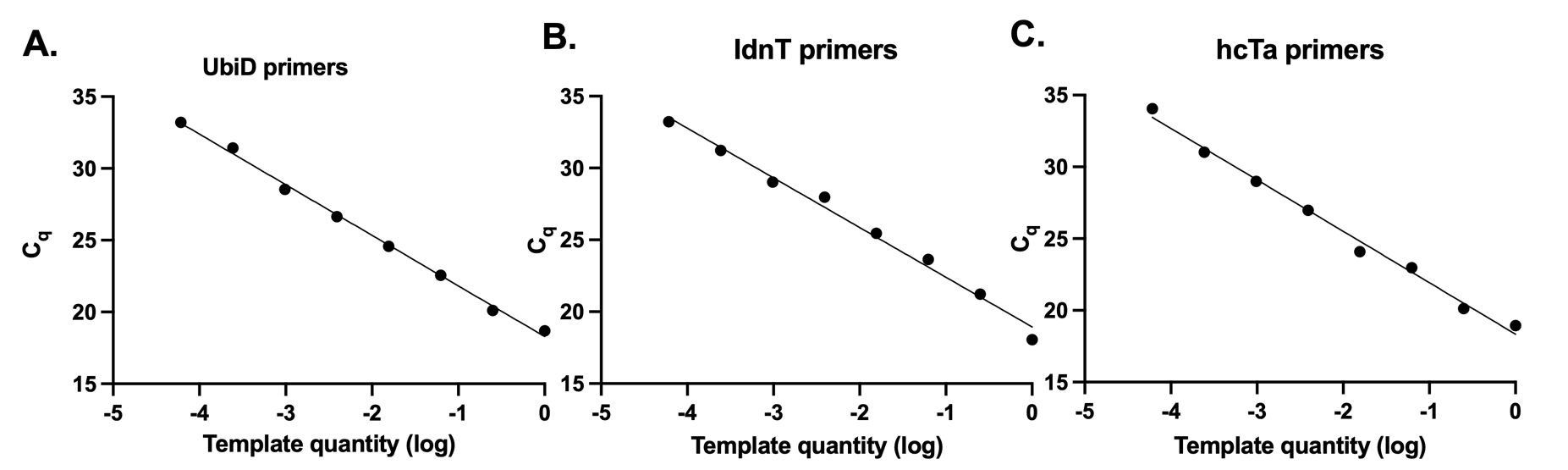 |
| --- |
| **Supplementary Figure 2.** Primer efficiency data of gene of interest (A). (UbiD) and housekeeping genes (B). idnT & (C). hcTA) in *E.coli* cells. |

**Supplementary Table 3.** Summary of data collection and refinement statistics of LpdD WT and variant structures

|  | **LpdD (Se-Met)** | **LpdD(Mn^2+^)***  **(PDB id: 8PO5 )** | **LpdD***  **(PDB id: 8PZO)** | **LpdD (H35A)***  **(PDB id: 8P4W)** | **LpdD (H61A)***  **(PDB id: 8PZH )** |
| --- | --- | --- | --- | --- | --- |
| **Wavelength (λ)** | 0.98 | 0.9798 | 0.9798 | 0.9798 | 0.9798 |
| **Resolution range** | 49.08-2.4  (2.486-2.4) | 74.99-2.2  (2.175-2.1) | 46.42  - 2.0  (2.072  - 2.0) | 37.32-1.78  (1.93-1.78) | 45.68-2.02  (2.092-2.02) |
| **Space Group** | I222 | I222 | I222 | P41212 | I222 |
| **Unit cells** | 53.3 92.8 125.4 | 53.5 92.98  126.82 | 53.1427 92.8494  125.009 | 58.99 58.99 83.54 | 52.7 91.58 124.69 |
| **a, b, c (Å)** | 90 90 90 | 90 90 90 | 90 90 90 | 90 90 90 | 90 90 90 |
| **Total reflection** | 331067 (34023) | 37104(3664) | 42645(4180) |  | 40410(3989) |
| **Unique reflection** | 12565 (1243) | 18833(1841) | 21329(2092) | 37230 (3227) | 20209(1995) |
| **Multiplicity** | 26.3 (27.3) | 2.0 (2.0) | 2.0(2.0) | 2.0(1.9) | 2.0(2.0) |
| **Completeness** | 99.9 (100.00) | 99.62(99.67) | 97.34(98.42) | 98.09 (83.59) | 97.45(77.92) |
| **Wilson B-factor** | 36 | 33.50 | 21.29 | 19.83 | 42.92 |
| **I/sI** | 8.67 (1.46) | 9.83(1.44) | 16.94(7.21) | 13.87(0.94) | 5.64(0.35) |
| **R_merge_** | 0.41 (2.2) | 0.07 (0.6) | 0.03(0.15) | 0.03(0.7) | 0.07(1.7) |
| **R-meas** | 0.42 (2.3) | 0.1(0.8) | 0.04(0.21) | 0.03(1.0) | 0.1(2.4) |
| **CC1/2** | 0.995 (0.574) | 1.0(0.9) | 1.0(0.9) | 1.0(0.5) | 1.0(0.5) |
| **Reflection used in refinement** | 12563 (1244) | 18827(1838) | 20765(2059) | 37094 (3097) | 19719(1560) |
| **Reflection used for**  **R-free** | 656 (79) | 877(88) | 1057(98) | 1993(167) | 1012(78) |
| **R_work_** | 0.21 (0.3) | 0.21(0.3) | 0.19(0.2) | 0.15(0.3) | 0.22(0.4) |
| **R_free_** | 0.25 (0.27) | 0.25(0.3) | 0.23(0.26) | 0.17(0.35) | 0.25(0.36) |
| **No. of non-hydrogen atoms** | 1903 | 1996 | 2137 | 1060 | 1799 |
| **macromolecules** | 1835 | 1854 | 1864 | 922 | 1726 |
| **Ligand** | 0 | 4 | 4 | 5 | 11 |
| **Solvent** | 68 | 138 | 269 | 133 | 62 |
| **Protein residues** | 240 | 242 | 246 | 121 | 232 |
| **RMS(bonds)** | 0.002 | 0.008 | 0.007 | 0.006 | 0.007 |
| **RMS (angles)** | 0.5 | 1.23 | 0.78 | 1.14 | 0.78 |
| **Ramachandran**  **favoured (%)** | 97.86 | 97.5 | 98.8 | 97.5 | 98.6 |
| **Ramachandran**  **allowed (%)** | 2.14 | 2.5 | 1.2 | 2.5 | 0.9 |
| **Ramachandran**  **outlier (%)** | 0.0 | 0.0 | 0.0 | 0.0 | 0.5 |
| **Rotamer outlier (%)** | 0.0 | 0.0 | 0.0 | 0.0 | 0.00 |
| **Clashscore** | 6.52 | 6.18 | 5.65 | 2.15 | 5.48 |
| **Av. B-factor (Å^2)^** | 42 | 37.1 | 32.3 | 25.8 | 53.5 |
| **Macromolecules** | 42 | 36.7 | 30.9 | 24.0 | 53.3 |
| **Ligand** | - | 51.1 | 58.4 | 32.8 | 87.3 |
| **solvent** | 45 | 41.7 | 41.3 | 38.3 | 54.4 |

*Each structure was determined from one crystal. Values in parentheses are for highest-resolution shell.
